# Decoding Liver Fibrosis: How Omics Technologies and Innovative Modeling Can Guide Precision Medicine

**DOI:** 10.3390/ijms26062658

**Published:** 2025-03-15

**Authors:** Gabriele Codotto, Benedetta Blarasin, Claudio Tiribelli, Cristina Bellarosa, Danilo Licastro

**Affiliations:** 1 Department of Life Science and Biotechnology, University of Ferrara, 44121 Ferrara, Italy; gabriele.codotto@unife.it; 2 AREA Science Park, 34149 Trieste, Italy; 3 Department of Life Science, University of Trieste, 34127 Trieste, Italy; benedetta.blarasin@fegato.it; 4 Fondazione Italiana Fegato ONLUS—Italian Liver Foundation NPO, 34149 Trieste, Italy; ctliver@fegato.it

**Keywords:** liver fibrosis, single-cell RNA-sequencing, spatial transcriptomics, iPSC-derived liver cells, organoids, PLCS, personalized medicine

## Abstract

The burden of chronic liver disease (CLD) is dramatically increasing. It is estimated that 20–30% of the population worldwide is affected by CLD. Hepatic fibrosis is a symptom common to all CLDs. Although it affects liver functional activities, it is a reversible stage if diagnosed at an early stage, but no resolutive therapy to contrast liver fibrosis is currently available. Therefore, efforts are needed to study the molecular insights of the disease. Emerging cutting-edge fields in cellular and molecular biology are introducing innovative strategies. Spatial and single-cell resolution approaches are paving the way for a more detailed understanding of the mechanisms underlying liver fibrosis. Cellular models have been generated to recapitulate the *in-a-dish* pathophysiology of liver fibrosis, yielding remarkable results that not only uncover the underlying molecular mechanisms but also serve as patient-specific avatars for precision medicine. Induced pluripotent stem cells (iPSC) and organoids are incredible tools to reshape the modeling of liver diseases, describe their architecture, and study the residents of hepatic tissue and their heterogeneous population. The present work aims to give an overview of innovative omics technologies revolutionizing liver fibrosis research and the current tools to model this disease.

## 1. Introduction: Liver Fibrosis

Chronic liver disease (CLD) includes a wide spectrum of diseases with diverse etiologies, ranging from metabolic disorders to viral infections, which can progress through inflammation, fibrosis, cirrhosis, and, eventually, hepatocellular carcinoma. Nowadays, CLD affects between 20 and 30% of the population worldwide [1].

Liver fibrosis is characterized by the accumulation of extra-cellular matrix (ECM) components to form scar tissue after chronic liver inflammation. This condition is the earliest symptom of worsening CLD, regardless of its etiology. There is still no resolutive therapy to treat the progression of the disease [2].

Liver fibrosis is a dynamic process resulting from the participation of nearly all hepatic cell types. When exposed to various stressors, hepatocytes undergo apoptotic death, resulting in the release of apoptotic bodies and immune cell recruitment. Kupffer cells (KCs) start releasing TGF-β and PDGF to stimulate quiescent hepatic stellate cells (HSCs) to differentiate into activated myofibroblasts, which eventually release ECM components (type I, II, IV collagen, entactin, laminin) and proinflammatory cytokines [3,4].

Although fibrotic tissue deposition affects liver function and homeostasis, liver fibrosis is still a reversible condition that can be treated by arresting fibrotic tissue deposition or reverting the differentiation of HSCs into myofibroblasts. All parenchymal and mesenchymal resident cells participate in this terrific process, with intriguing discoveries revealing the role of immune cells in interacting with the parenchymal components of the liver [5].

The important contribution of the different cells in liver fibrosis underlines the complexity of the disease and the necessity of diverse approaches to study the disease from the molecular point of view. Cell heterogeneity is an extremely challenging factor due to the multifaceted roles of mesenchymal components and the responses of the parenchymal counterpart.

The intricate architecture of the liver is another fascinating aspect to be unraveled. The outstanding organization of hepatic cells into liver units, known as lobules, holds the key to all the molecular mechanisms underlying the functional activities of the liver. Moreover, the immune cell landscape within the liver is increasing the complexity of the system [6].

Mankind has always been intrigued by the liver; the Etruscan haruspex used it to interrogate fate to get good omens and predict the future. In more recent times, the perspective has shifted. With the advanced progress of the omics field, we are looking for a broader range of information to investigate the molecular mechanisms involved in liver homeostasis, regeneration, and diseases.

In-vitro and ex-vivo models aim to unravel the processes involved in hepatic diseases [7].

## 2. Unraveling the Molecular Diversity of Liver Fibrosis: Single-Cell and Spatial Transcriptomics

The emerging field of omics approaches for the study of liver pathophysiology is growing daily due to the information it can provide and the wide range of possible applications [8].

Omics refers to the global, large-scale study of different biology fields, including genomics, epigenomics, transcriptomics, proteomics, and metabolomics. The power of this frontier resides in the large amount of information that can be derived from a single experiment and the sensitivity of the methods used.

Transcriptomics is the comprehensive study of the RNA molecules that are present in the cell at a specific moment of cell life. Techniques such as microarrays, serial analysis of gene expression (SAGE), and RNA-sequencing (RNA-seq) were the first techniques used to detect and analyze the transcriptome. The field has recently developed with the implementation of single-cell RNA-sequencing (scRNA-seq) and its variant, single-nucleus RNA-sequencing (snRNA-seq), and spatial transcriptomics (ST) [8,9].

Unlike bulk RNA-seq, single-cell technologies can provide information about the transcriptomics of each cell that is present in the sample, allowing the investigation of cell heterogeneity and the interplay with other cell components and analyzing the molecular diversity of the liver.

**Figure 1:**
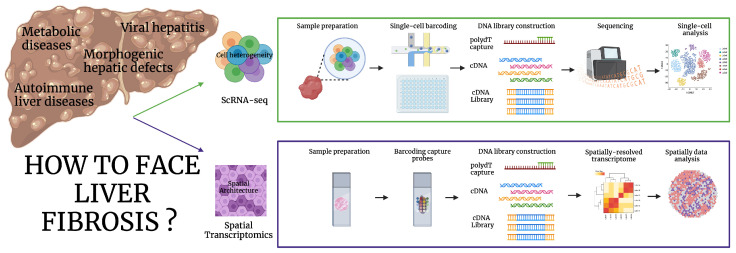
Strategies to study liver fibrosis. Liver fibrosis is a common outcome to many etiologies. Cell heterogeneity can be investigated by single-cell RNA-sequencing (scRNA-seq), while spatial architecture can be studied by spatial transcriptomics (ST). From the homogenized tissue, a single-cell suspension is barcoded, and RNA is captured to generate cDNA and DNA libraries. The libraries are deep sequenced to decode single-cell transcripts and population heterogeneity. The samples for ST are laid out on the grid of a glass covered with polydT barcode probes, the transcripts are then released, and cDNA libraries are constructed and sequenced to create a spatially resolved map. Created in BioRender. Codotto, G. (2025) https://BioRender.com/l60i579.

Furthermore, the combination of ST and scRNA-seq liver architecture can be used in a very informative way. Refer to Figure 1, which summarizes the two main focuses of liver fibrosis research and the methods to investigate it.

For a detailed overview of the technologies currently available on the market, we strongly recommend referring to the detailed review by Wang [10].

Recently, the hepatology community has shown a growing interest in the functional zonation of hepatocytes, a key feature in liver architecture.

Moreover, when functional zonation of hepatocytes is disrupted by non-physiological mechanisms, hepatocyte behavior is altered, leading to the onset of metabolic dysfunction-associated steatotic liver disease (MASLD) and liver fibrosis [11].

To investigate hepatocyte zonation, scRNA-seq and ST revealed key metabolic mechanisms occurring within hepatocytes, providing valuable insights into the spatial architecture [12,13].

### 2.1. ScRNA-Seq

The complexity of liver tissue architecture arises from the diversity of the resident cells that populate the tissue. The parenchymal fraction is represented by hepatocytes and ductal cells (cholangiocytes), while the mesenchymal component is represented by immune-inflammatory cells, liver endothelial cells, and HSCs.

Each cell type is further represented by different subpopulations characterized by specific transcriptomic signatures. Through scRNA-seq, liver zonation has been further unraveled, leading to a better comprehension of the specific roles of each cell subpopulation in both healthy and diseased tissues [14].

The necessity to profile each cell subpopulation led Andrews and colleagues to identify, through scRNA-seq and snRNA-seq 20, distinct cell populations that reside in specific areas of a healthy liver [15]. While scRNA-seq can be biased by sample preparation steps, snRNA-seq offers improved profiling of each cell subpopulation, as it does not require tissue digestion or mechanical disruption.

The preparatory phases mostly affect the final yield of cells sequenced for the transcriptomic characterization. In their study, Andrews and colleagues demonstrated that snRNA-seq ensures the detection of additional cell types, belonging particularly to the mesenchymal fraction. Due to their resistance to dissociation techniques, the cell-type frequencies of mesenchymal cells are distorted in scRNA-seq. Since the snRNA-seq protocol excludes the portion of cytoplasmic transcripts, such as ribosomal and mitochondrial fractions, the difference in gene diversity is given by the proportion of unique molecular identifiers in scRNA-seq [15]. ScRNA-seq was used to deconvolve mesenchymal cell heterogeneity in mouse healthy and fibrotic liver and revealed the spatial zonation of HSCs. Two topographically distinct hepatic stellate cell populations were identified based on their transcriptomic signatures: portal vein-associated HSCs and central vein-associated HSCs (CaHSCs).

Furthermore, in a CCl_4_-induced fibrotic mouse model, CaHSCs were responsible for a wide pathogenic collagen production and deposition [14], leading to the conclusion that they are the main subtypes involved in centrilobular liver injury, which subsequently leads to liver fibrosis.

### 2.2. Spatial Transcriptomics

The changes in the liver tissue architecture result from the activity and interaction of the cellular components that populate the liver.

A challenging issue is the extreme variability of the fibrotic liver; different regions present distinct features in terms of immune cell infiltration and inflammation, collagen deposition, and tissue stiffness.

Immune cells are the main players in initiating the inflammatory response that leads to the activation and transdifferentiation of HSCs. After the induction of this process, activated HSCs change their functional roles towards the collagen-secreting myofibroblast phenotype, leading to collagen deposition.

Liver tissue architecture is irreversibly altered by the massive deposition of fibrotic tissue, with certain regions being more affected than others while some regions retain normal liver features.

ScRNA-seq provides both qualitative and quantitative information about parenchymal and non-parenchymal cell populations, but it still lacks spatial information.

The joining link to complement spatial information and quantitative single-cell transcriptomics information is offered by ST technologies.

The fibrotic liver is highly heterogeneous in terms of fibrotic tissue deposition and inflammatory cell distribution, not only across samples from different patients, but also within different regions of the same tissue.

To investigate fibrotic liver tissue architecture, Chung and colleagues showed that transcriptomic profiles of fibrotic regions differ significantly among distinct areas of the same cirrhotic liver, and conventional histopathology correlates with ST results. ST studies demonstrated higher frequencies of 57 upregulated gene signatures in fibrotic tissue, corresponding to four biological processes: RNA translation, antigen presentation, fibrogenesis, and immune function. Furthermore, non-fibrotic areas of the tissue showed enriched gene signatures belonging to hepatocytes, cholangiocytes, and cycling cells. In contrast, gene deconvolution of the fibrotic regions revealed a significant increase in the presence of immune infiltrating cells [16].

Another study by Li underlined the transcriptional features of metabolic dysfunction-associated steatohepatitis (MASH)-related fibrosis.

From a cellular point of view, MASH samples present a relevant increase in myofibroblasts compared to HSCs. Notably, cell communication was led by collagen-related pathways together with additional pathways, including FN1, LAMININ, PTPRM, THBS, and VISFATIN. Transcriptional signatures in MASH tissue were analyzed through scRNA-seq and ST, revealing six genes with either high or low expression in the fibrotic regions of MASH livers. While ADAMTSL2, PTGDS, and S100A6 positively correlated with the presence of fibrotic tissue as assayed by Haematoxylin and Eosin staining, PPP1R1A, ASS1, and G6PC expression was considerably lower as the degree of fibrosis increased. Interestingly, together with the spatial profile, the pseudo-temporal evolution of fibrosis was determined, starting from a baseline condition that considered hepatocytes and their transcriptional evolution status, followed by the further recruitment of T cells, KCs, and HSCs, and culminating in the final conversion into myofibroblasts [17].

## 3. Unraveling the Molecular Mechanisms Involved in Liver Fibrosis: Gold Standard and Emerging 2D In Vitro Models

### 3.1. Primary and Immortalized Cell Lines

HSCs are the main actors playing a role in liver fibrosis. Primary HSCs are activated by TGF-β signaling, which triggers progressive cellular changes leading to an HSC-to-myofibroblast-like destiny.

After 15 days of in vitro culture, HSCs acquire a characteristic morphology that is very different from the physiological one. More specifically, at day 0, they present an oval-shaped morphology that progressively changes into a star-shaped form, accompanied by the presence of pseudopodia [18]. The fully activated HSCs assume a myofibroblast-like shape after 14 days in culture. The gradual change into myofibroblast-like cells affects not only the conformation and the structural features of the HSCs but also the functional activities of the cell.

Due to these deep changes, myofibroblast-like cells can secrete a massive amount of extracellular matrix, including collagen and ECM proteins.

Since primary HSCs in 2D cultures develop and grow on a flat surface, they lack cell-ECM interactions. The absence of 3D structural conformation deeply affects the functional activity of primary cells, which will irreversibly lose in situ cellular features over time. As a result, they can be maintained only for a short-term culture.

For the establishment of 2D-cell culture, primary HSCs have been sourced from the livers of humans, rats, and mice [19,20].

Despite several limiting conditions of primary cell culture, HSCs are still considered the gold standard model for investigating HSC biology and liver fibrosis-promoting events [21].

Liver fibrosis is a complex condition that is enhanced by persistent inflammation and fibrotic tissue deposition with a contribution of different hepatic cell types [18].

The inflammation and matrix deposition processes are triggered and supported by different liver resident cells. Therefore, a single primary cell line cannot fully mimic in vitro the pathological mechanisms responsible for liver fibrosis development.

As a consequence, the lack of cellular heterogeneity may provide a misleading vision of the complex processes driving liver fibrosis [21].

Despite several disadvantages affecting the 2D culture of HSCs, the easy culture conditions and the physiological state in the early days of culture make primary HSCs a valuable tool to perform drug testing and disease modeling. Because of the difficult accessibility to human liver samples, the availability of primary human HSCs is likely compromised. An alternative model for studying HSC biology is the use of immortalized HSCs lines, including LX-2 cells [22,23].

On the one hand, this model offers significant advantages due to its high scalability, low costs, and the preservation of the same transcriptional signature as primary HSCs. On the other hand, LX-2 maintains the fully activated state in the myofibroblast-like cell state. The lack of different stages of differentiation of primary HSCs during the inflammatory-fibrotic process is a limitation that can be addressed only using a multicellular model comprehensive of different liver resident cells [24].

### 3.2. iPSC-Derived Hepatic Cells

Liver complex architecture is finely organized into a portal-central axis, which is populated by specific cellular subtypes in each area of its structure.

**Figure 2:**
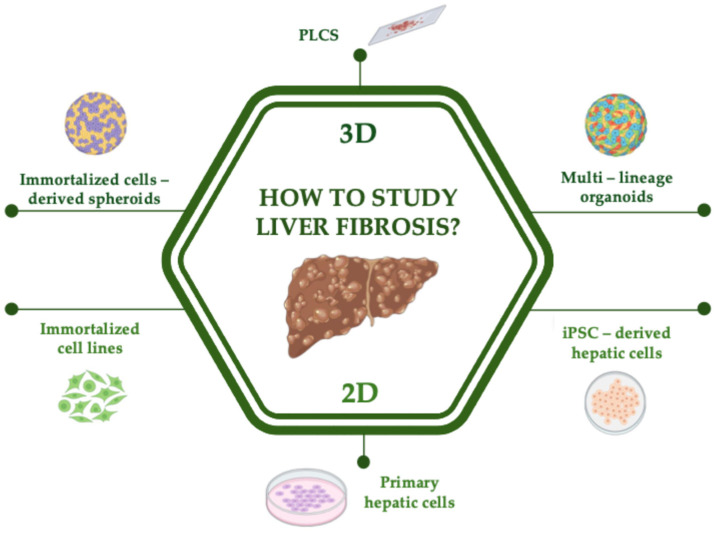
Scheme of in vitro and ex vivo available models to investigate liver fibrosis. The 2D models include immortalized cell lines, induced pluripotent stem cells (iPSC)-derived hepatic cells, and primary hepatic cell lines, while the 3D models include immortalized cell-derived spheroids, multi-lineage organoids, and precision-cut liver slices (PLCS). Created in BioRender. Codotto, G. (2025) https://BioRender.com/l60i579.

Due to the complexity of liver architecture and heterogeneous cell populations, researchers tried to study liver fibrosis using different model systems and approaches. The main models are illustrated in Figure 2.

Each cell type has a different state depending on its localization along the portal-central axis, a phenomenon known as spatial zonation [11].

The challenge of precisely replicating the complex tissue structure in vivo led many researchers to generate cell models starting from iPSCs, aiming to develop different hepatic cell components by modeling the same stages of embryonic development.

In the early development stages, the blastocyst presents the outer cells, committed in extra embryonal fates. The inner cell mass (ICM) is responsible for the generation of all the tissues of the embryo. Starting from ICM, embryonic stem cells (ESCs) can be sourced and used to further generate organoids [25].

At the gastrulation stage, mesenchymal to epithelial transition leads cells to migrate following precise signaling pathways. Strict transcriptional programs lead the gastrula to divide into three germ layers: ectoderm, mesoderm, and endoderm. Each of these structures plays a crucial role in the development of specific organs.

The gastrointestinal tract is derived from the endodermal layer in response to precise morphogenic signals that orchestrate liver development.

Moreover, liver formation is triggered by the expression of posterior morphogenic factors, which confer a posterior identity to the embryonic tissue.

Signaling factors, including Wnt family factors, BMP4, FGF, and retinoic acid, are responsible for progressive differentiation stages in which the foregut is further differentiated into hepatic progenitor stage (hepatoblast) and, eventually, into hepatic-like cells [25].

The term “hepatic-like cell” refers to iPSC-derived cells that resemble hepatic cells in their functional activities, maintaining similar genomic and transcriptional signatures. Unfortunately, the differentiation stages affect the genomic and transcriptional states of the differentiated cells. These events limit the complete maturation of the cells, which continue to express fetal markers [26].

Many issues emerged concerning the use of ESCs, primarily due to ethical reasons. As a result, many countries have prohibited the use of ESCs for research purposes [27].

To address this issue, in 2006, Yamanaka described, for the first time, the way to reprogram mouse fibroblasts into iPSCs by introducing, through transfection, four factors (Oct3/4, Sox2, c-Myc, and Klf4) [28].

Recently, iPSC technology has gained significant popularity due to its ability to maintain pluripotency and be directed toward various fates for different applications [29].

The possibility to reprogram differentiated cells of the patients into iPSCs preserves the patient’s genetic background and allows an easy access to the samples. Regardless of the pathology, obtaining primary tissues from patients is a challenging issue due to the invasive nature of the procedures, such as biopsy or surgery, required to collect samples. iPSCs can be obtained from nearly all cell types. In addition, the procedures to obtain fibroblasts, blood, or urine samples are painless and easy and can also be repeatedly performed without impacting the patient’s health.

Based on the growth factors that orchestrate each stage of embryonic development, many research groups put a great effort into optimizing the culture conditions that lead iPSCs toward different hepatic cell specifications.

The group of Sancho-Bru developed hepatic stellate-like cells starting from human-induced pluripotent stem cells (hiPSC) by inducing mesodermal fate. The culture medium was enriched with growth factors belonging to the TGF-β family, including BMP or activin A, together with mitogenic growth factors such as FGF. hiPSC-derived HSCs resemble transcriptional, cellular, and functional activities as primary HSCs. Their functional state, according to the gene expression profile, was intermediate between the quiescent and the activated state of myofibroblasts. After 3D co-culture with HepaRG hepatocytes, the iPSC-derived HSCs showed fibrogenic activity. This model provides a useful tool for investigating HSC biology and the molecular events of liver fibrosis [30].

## 4. Unraveling the Molecular Mechanisms Involved in Liver Fibrosis: Gold Standard and Emerging 3D In Vitro Models

### 4.1. Immortalized Cell Lines Derived 3D-Models

Given the many advantages of immortalized cell lines, many protocols for the generation of a 3D spheroid model have been published. Multilineage 3D spheroids were developed by Pingitore et al. to investigate the onset of liver fibrosis by inducing steatosis through the administration of fatty acids followed by inflammation and fibrosis stages. Two cell lines, LX-2 and HepG2, were cocultured in a 3D matrix to assemble functional spheroids capable of sustaining both steatotic and fibrotic processes. The precise ratio of each cell population was fundamental for the proper formation of the spheroid. For this reason, we can conclude that this model is more constructed rather than naturally developed [31].

Brenner et al. described a similar model of spheroid obtained from the co-culture of primary hepatocytes and primary HSCs. While alcohol administration to the spheroids mimicked metabolic dysfunction and alcohol-related liver disease, fatty acids administration showed the development of MASH and, eventually, liver fibrosis. As expected, after the induction of the fibrogenic stimulus, spheroids showed enhanced expression of fibrogenic genes (SERPINE1 and TIMP1) and proteins (Collagen Type I, αSMA) [32].

### 4.2. iPSC-Derived Multilineage Organoids

The power of genetic reprogramming opens new horizons in regenerative medicine. iPSC, once differentiated, can give birth to a lot of different cell types [33]. This great advantage has been demonstrated by Ramli and colleagues, who developed a hepatobiliary organoid (HO) model composed of hepatocyte and cholangiocyte-like cells. The optimization of the culture conditions allows the co-existence of more than only one cell type. Unsurprisingly, multicellular organoids can be created and maintained in culture by retracing developmental stages [34].

Ramli’s organoid model showed the ability to mimic the physiological features of hepatocytes and cholangiocytes, as well as functional bile ducts, which were disrupted after the treatment with troglitazone.

Furthermore, HOs showed structural features tested by live imaging. Functional activity assayed by albumin and apolipoprotein B expression, together with gamma-glutamyl transferase and alkaline phosphatase expression. Ramli used the same HO model to study hepatocellular steatosis induced by stimulation with free fatty acids (FFAs) [35].

Although this model cannot replicate the fibrotic process due to the lack of HSCs, it offers interesting opportunities to study lipid accumulation and MASLD, which are a prelude to the triggering events involved in liver fibrosis.

Ouchi and colleagues developed multicellular human liver organoids (HLOs) to study hepatocellular steatosis and liver fibrosis. HOs were generated by retracing developmental stages from iPSC to definitive endoderm, followed by progression to the foregut, and eventually differentiated into HLOs [36].

HOs are mainly composed of hepatocyte-like cells, stellate-like cells, and Kupffer-like cells, three of the major cell types involved in MASLD. ScRNA-seq analysis revealed five distinct cell clusters, including bile duct bipotent stem cells and hepatocyte-like cells, which showed periportal vein markers, while the expression of central venous markers was minimal.

After treatment with FFAs, HLOs maintain a similar profile as the tissue in vivo. Ballooning and lipid deposition were assessed by immunofluorescence, and the results showed a dose-dependent trend.

After FFA treatment, HLOs recapitulate the inflammatory condition following lipid intake and accumulation, resulting from the contributions of various hepatic cell types. Lipid accumulation drives Kupffer-like cells to upregulate TNFα and IL-8 expression levels. Considering HOs, flow-sorting experiments individuated Kupffer-like cells as the main ones responsible for cytokine secretion among the different cells composing HOs.

Surprisingly, together with lipid accumulation and inflammation, HOs were also capable of modeling liver fibrosis in a realistic, pathological way, as assessed by increased levels of α-SMA and vimentin, P3NP, and collagen deposition.

A correlation between the stiffness of HLOs treated with FFAs and those treated with LPS was demonstrated using atomic force microscopy (AFM). New insights into the readout of human liver fibrosis demonstrated that the fibrosis score appears comparable when modeling liver fibrosis with HLOs and in in vivo conditions, despite the causative event [36].

Recently, Takebe et al. developed a further sophisticated model of iPSC-derived liver organoids that recapitulates the zonal liver architecture in vitro. As previously mentioned, liver zonation is intriguingly involved in homeostasis and disease. Hepatic progenitors have been cultured in conditions enriched with ascorbic acid and bilirubin, two factors that influence liver zonation fate and metabolic processes. SnRNA-seq mapped hepatoblast differentiation trajectory from a transcriptomic perspective, mimicking the same organization present in the human liver. Furthermore, HOs were transplanted into recipient rats that underwent bile duct ligation, resulting in ameliorating the hepatic condition of the injured rats [37].

To study abnormal bile ducts and associated liver fibrosis, Guan and colleagues employed an iPSC-derived hepatic organoid model, previously developed by their group to characterize monogenic disorders. The ARPKD gene was mutated through the CRISPR-Cas9 gene editing technique. Autosomal Recessive Polycystic Kidney Disease (ARPKD) is a monogenic disease that primarily affects the kidney but also has a relevant outcome on the liver. The main hepatic dysfunctions are dilated intrahepatic bile ducts and congenital liver fibrosis. The organoid cell composition was characterized with scRNA-seq analysis, revealing a mix of hepatocyte-like cells, ductal-like cells, and bi-potential progenitor cells, as well as endothelial and stellate-like cells [38].

Multicellular organoids were subjected to liver fibrotic progression response because of the lack of the ARPKD gene. Increased amounts of collagen fibers were detected throughout the entire organoid. ARPKD organoids showed a higher accumulation of collagen and ECM proteins when compared to healthy controls. The deposition of collagen fibers and the stiffness of the tissue were considered indicative factors in characterizing fibrosis severity [39].

While Ouchi characterized the fibrosis rate with AFM [36], Guan analyzed liver fibrosis through second harmonic generation microscopy [39]. As previously shown by Ouchi, Guan also established a correlation between liver fibrosis severity in hepatic tissue and the one observed in the organoids.

## 5. Unraveling the Structural and Architectural Features Involved in Liver Fibrosis: Modeling Liver Fibrosis with Ex Vivo Models

### Precision-Cut Liver Slices

Although HSCs are still considered the gold standard for the study of the activation and progression phases of liver fibrosis, the presence of a single cell type limits the study of HSCs biology, not allowing a global vision of the intercellular interactions present in the tissue in vivo.

For a deeper comprehension of these interactions, precision-cut liver slices (PCLS) offer a well-established tool for studying the molecular mechanisms of the disease and testing of anti-fibrotic drugs.

PCLS maintains the complex architecture and cell heterogeneity of the liver, including cell-to-cell and cell-to-ECM interactions.

The preparation of PCLS requires specific instruments, including a tissue slicer, a mechanical drill with a hollow bit, and a specific incubator [40]. The slices usually measure 8 mm in diameter and 250 µm in thickness, dimensions that ensure sufficient nutrient supply and gas exchanges to the whole tissue, including the inner layers [41].

To demonstrate the functionality of PCLS, the ATP rate was evaluated at 48 to 96 h [42], showing normal levels. Unfortunately, after this short period, cells start to lose their functionality, and the tissue structure loses its physiological features.

The first liver PCLS has been prepared starting from rats [43]. Fibrotic liver slices were obtained from rats that underwent bile-duct ligation and showed an increase in the key marker expression of liver fibrosis. α-SMA and procollagen 1a1 mRNA expression was consistently increased in animals that underwent surgical bile duct ligation. To assess anti-fibrotic drug effects, Gleevec, pentoxifylline, and dexamethasone were added to the incubation medium, resulting in decreased α-SMA and pro-collagen 1a1 mRNA levels [43].

In the following experiments, Olinga and colleagues moved to human tissues and performed similar experiments with consistent results.

After human liver slices were treated with CCl_4_, cell viability decreased, and early markers of HSC activation (HSP47 and αβ-crystallin) were assessed. After prolonged incubation with CCl_4_ (24 h), levels of procollagen 1a1 and α-SMA were measured, showing an increase in mRNA expression [42].

Interestingly, Mabire and colleagues used human and murine PCLS, unraveling the role of mucosal-associated invariant T (MAIT) cells in the progression of end-stage liver fibrosis. Acetyl-6-formylpterin was shown to inhibit MAIT cells, limiting the expression of the Ly6C^lo^ phenotype of monocyte-derived macrophages and, eventually, preventing the progression of liver fibrosis [44].

Wang and colleagues evaluated the treatment of erlotinib in different murine cirrhotic models (choline-deficient, L-amino acid-defined, high-fat diet, thioacetamide, diethylnitrosamine, and carbon tetrachloride) and reported that erlotinib significantly reduced profibrogenic gene expression. Furthermore, they treated PCLS from normal mice and showed that TGF-B1-upregulated expression of the Acta2 gene in HCSs was inhibited by erlotinib treatment. Additionally, the results were corroborated with human cirrhotic PCLS [45].

A recent study investigated the therapeutic effect of naringenin (NRG), asiatic acid (AA), and icariin (ICA) as antifibrotic drugs. Both mouse PCLS (mPCLS) and cirrhotic human PCLS (chPCLS) were treated with the compounds, showing that NRG reduced COL1A1 expression in both mPCLS and chPCLS in a concentration-dependent trend. IL-1β and TNF-α protein and gene expression were inhibited by NRG in mPCLS, while IL-1β gene expression was inhibited in chPCLS. Additionally, AA reduced COL1A1 and PCOL1A1 in chPCLS, while ICA downregulated Col1a1 and Acta2 gene expression [46].

Although this model cannot be cultured for a long time, it represents a reliable tool to investigate liver fibrosis biology due to its capacity to maintain structural and architectural features.

## 6. Future Perspectives: Organoids and Omics as a Combined Tool for Precision and Translational Medicine

The dramatic global burden of CLD created a growing interest in studying liver fibrosis as the first symptom of the progression of liver diseases. Understanding the molecular mechanisms that trigger and induce liver fibrosis currently remains fundamental both for basic and translational research.

Cutting-edge in vitro and ex vivo models, such as organoids, offer great advantages for studying and modeling liver diseases, providing a valuable platform for drug testing and genetic screening. The potentials, limitations, and applications of these model systems for investigating liver fibrosis are summarized in Table 1.

The scarce availability of primary liver tissue from patients is a limiting factor for the study of hepatic diseases. Unsurprisingly, the outstanding potential of iPSC to be differentiated into HOs allows the obtaining of hepatic organoids with the same genetic background of the patient. Hence, HOs have already been used as a solid instrument for personalized translational medicine [47,48].

If iPSC technology addresses the issues related to sample scarcity, the advent of genome editing technologies, such as clustered regularly interspaced short palindromic repeats/Cas9 (CRISPR/Cas9), allows us to correct genetic mutations of patient-derived iPSC. The insertion of a mutated gene recreates the phenotype of the monogenic disease in the organoid model [49].

Guan and colleagues utilized piggyBac transposon and CRISPR/Cas9 to achieve genome editing of iPSC lines, introducing a homozygous ARPKD mutation (PKHDI^36Met^) and, eventually, obtaining multicellular organoids harboring ARPKD mutation. Interestingly, ARPKD mutation was significantly altering the function of hepatic cells. ARPKD cholangiocytes showed altered polarity and less maturity driven by TGF-β pathway alteration, while activated STAT3 signaling pathway and PDGFRB higher expression were described in collagen-producing myofibroblasts. In conclusion, STAT3 and PDGFRB play an important role in the pathogenesis of liver fibrosis [39].

Choi and colleagues studied dyskeratosis congenita (DC), a multiorgan disease caused by a defect in the DKC1 gene. Regarding the phenotypic effect on the liver, mutations in the DKC1 gene affect telomerase activity, leading to liver fibrosis. In this study, Choi and colleagues obtained iPSC from patients. The mutation of DKC1 was corrected with CRISPR/Cas9 technology to obtain isogenic iPSC and further generate admixed iPSC-derived hepatic organoids [50].

The outstanding power of CRISPR/Cas9 technology is driving its expanding use in genetic screening and in the correction or introduction of genetic mutations in cellular models. As this is not the primary focus of this review, we refer to Ramakrishna, who elegantly summarized the advantages, limitations, and potential applications of genome editing techniques [51].

The integrative approach between omics technologies and organoid models opens new horizons to understand the molecular insights of the disease, offering the advantage of studying disease conditions in vitro using reliable models.

Takebe and colleagues employed their multi-lineage organoid model to integrate organoid modeling and clinical analysis for precision hepatology. In their research, they investigated genetic contribution in MASLD with an en masse phenotyping approach, which focuses on unraveling the individual genetic differences between 24 patients affected by MASLD. The concept of an “organoid village” has been introduced to describe organoid co-culture derived from different patients [48].

This in-a-dish genome-wide association study appears to be a winning strategy to unravel personalized phenotypes in a collection of cells derived from different donors. The advantage of this in vitro approach resides in the absence of non-genetic players that could act as confounding factors when analyzing the genetics of complex metabolic diseases. Diet and environmental factors play a decisive role in the investigation of these diseases. While in vivo models are affected by external factors, in vitro systems lack this effect and represent a realistic tool for the study of genetic components [52].

Yasmine and colleagues investigated the role of the ACMSD gene in the development of MASLD. They mimicked the disease using different model systems, including Hos, and demonstrated that inhibiting the gene led to improvements in inflammation, fibrosis, and lipid accumulation, confirming the role of ACMSD as a key gene involved in MASLD [53].

Remarkable considerations should be pointed to the role of single-cell techniques in the context of organoid screening. ScRNA-seq allowed the underlining of the transcriptomic similarity between the tissue and organoids derived from the patients. ScRNA-seq and ST are invaluable tools for investigating cell identity and histoarchitecture. When integrated, this information accelerates the understanding of the transcriptomic landscape at the basis of liver fibrosis.

Furthermore, emerging omics approaches have already been used to investigate the methylome in liver diseases and transplantation studies [54].

Liquid biopsy is an emerging tool to predict the outcome of liver diseases without the need for tissue biopsy. The next frontiers aim to perform multiomics in liquid biopsy for early prediction of the diseases [55].

Furthermore, new omics techniques, including high throughput proteomics [56] and epigenomics [54], could give a wider spectrum of the pathologic condition, opening the way to integrated system biology approaches.

## 7. Conclusions

The study of liver fibrosis should address cellular heterogeneity and liver architecture.

Single-cell technologies and ST are challenging technologies to unravel liver fibrosis complexity and the immune landscape regulating the fibrotic processes.

The insights gained from the output of this technology are twofold: it provides information on the cellular components and their spatial organization into the tissue, as well as on cell heterogeneity, which is validated to investigate the different lineages of the same hepatic cell type.

Although PLCSs face a challenge regarding the availability of primary tissue, they can address many questions related to liver architecture. The iPSC milestone discovery inaugurated a new era in cell biology, allowing the development of the organoid field while breaking down the limitations connected to primary tissue need. Moreover, the possibility of recreating the pathophysiology of the liver *in-a-dish* with human cellular model systems paves the way for precision and regenerative medicine.

Multilineage organoids mimicking the human liver can give further information not captured by organoids composed of a single cell type. Additionally, HOs ensure an optimal platform for genetic screenings and drug testing, and a co-culture enrichment with peripheral blood mononuclear cells can recreate the best conditions to study liver fibrosis.

Tissue avatars, such as organoids, together with the advantages of liquid biopsy, are driving medicine towards a personalized therapy approach, where treatments are tailored to individual patients.

## Figures and Tables

**Table 1:** Potentials, limitations, and applications of in vitro and ex vivo model systems.

Cell Model	Potentials	Limitations	Applications	References
Primary hepatic stellate cells	Gold standard to study the activation stages of hepatic fibrosis	Single cell type	Study of molecular mechanism of hepatic fibrosis	[19,20]
		Limited availability	Drug testing	
		Can be cultured for few days		
Cell lines	Single cell type	Derived from transformed or immortalized tumoral cell lines	Study of molecular mechanisms of hepatic fibrosis	[22,23]
	CRISPR—Cas9 genome editing	Genetic and chromosomal aberration		
	Resemble the activated phenotype of myofibroblasts	Single cell type		
	Reproduce liver fibrosis mechanisms			
induced pluripotent stem cells (iPSC)—derived hepatic cells	Maintain cell functional activities and genetic identity	Lack full maturity	Study of molecular mechanism of hepatic fibrosis	[30]
	Starting material easily available	Challenging technology		
	Genetic reprogramming through CRISPR—Cas9		Drug testing	
Immortalized cell lines—derived 3D models	Assembled, not developed Easy to generate Long-term culture	Lack 3D structure	Study of molecular mechanism of hepatic fibrosis	[31,32]
		Genetic and genomic aberrations	Drug testing	
induced pluripotent stem cells (iPSC)—derived multilineage organoids	Cell heterogeneity	Lack full maturity	Study of molecular mechanism of hepatic fibrosis	[35,36,38,39]
	Multi-cell type	Cell line dependency	Drug testing	
	Long-term culture Genetic reprogramming (CRISPR-Cas9 technology)	Complexity in generation and maintenance	Metabolic studies	
Precision—cut liver slices	Tissue architecture maintained	Short-term culture	Liver architecture study	[40,42,43]
			Drug testing	
	Cell heterogeneity maintained			
	Same genetic background of the patient			

## Abbreviations

The following abbreviations are used in this manuscript:

CLD	Chronic liver disease
iPSC	Induced pluripotent stem cells
ECM	Extra-cellular matrix
KCs	Kupffer cells
HSCs	Hepatic Stellate cells
SAGE	Serial analysis of gene expression
RNA-seq	RNA-sequencing
scRNA-seq	Single-cell RNA sequencing
snRNA-seq	Single-nucleus RNA sequencing
ST	Spatial Transcriptomics
MASLD	Metabolic dysfunction-associated steatotic liver disease
CaHSCs	Portal vein-associated HSCs
MASH	Metabolic dysfunction-associated steatohepatitis
ICM	Inner cell mass
ESCs	Embryonic stem cells
hiPSC	Human induced pluripotent stem cells
HO	Hepatobiliary organoid
FFAs	Free fatty acids
HLOs	Human liver organoids
AFM	Atomic force microscopy
ARPKD	Autosomal recessive polycystic kidney disease
PCLS	Precision-cut liver slice
MAIT	mucosal-associated invariant T
NRG	Naringenin
AA	Asiatic acid
ICA	Icariin
mPCLS	Mouse precision-cut liver slice
chPCLS	Cirrohotic human precision-cut liver slice
DC	Dyskeratosis congenita
